# A Thematic Analysis of Sleep Behavior Self-Regulation in Young Adults with Type 1 Diabetes

**DOI:** 10.3390/diabetology7040080

**Published:** 2026-04-14

**Authors:** Madeline Long, Dayna A. Johnson, Youjeong Kang, Stephanie Alisha Griggs

**Affiliations:** 1 Emory University, Nell Hodgson Woodruff School of Nursing; 2 Emory University, Department of Epidemiology, Rollins School of Public Health; 3 Emory University, Nell Hodgson Woodruff School of Nursing; 4 Emory University, Nell Hodgson Woodruff School of Nursing

**Keywords:** type 1 diabetes, sleep, self-regulation, young adults, thematic analysis, qualitative

## Abstract

**Background/Objectives:**

Sleep is critical for young adults, particularly those with type 1 diabetes (T1D), who face unique challenges in achieving recommended sleep and diabetes health targets. The purpose of this study guided by the theoretical framework of self-regulation theory is to explore how these individuals navigate self-regulatory processes in their sleep behaviors through mechanisms of self-monitoring, self-judgment, and self-evaluation.

**Methods:**

A qualitative descriptive design was implemented using semi-structured interviews with 34 young adults (ages 18–30) living with T1D. Data were collected through sleep diaries, actigraphy, and continuous glucose monitoring, followed by thematic analysis to identify sleep behavior self-regulation patterns.

**Results:**

Three primary themes were identified: (1) Sleep Behavior Self-Monitoring – highlighting participants’ awareness of their sleep habits and the diabetes-related impacts on these habits; (2) Self-Judgment – reflecting how personal and societal standards inform their evaluation of sleep health; (3) Self-Evaluation – showing emotional responses associated with sleep outcomes, where good sleep led to positive feelings and motivation, while poor sleep resulted in frustration.

**Conclusions:**

Understanding sleep behavior self-regulation among young adults with T1D is crucial for improving sleep health and diabetes management. Targeted interventions incorporating sleep education and self-regulatory strategies may enhance both perceived sleep quality and overall well-being in this population.

## 1. Introduction

Sleep is a foundational health behavior that plays a critical role in physical, cognitive, and emotional functioning [1–4]. For young adults, sleep that is of adequate quantity and quality is essential for academic performance, occupational functioning, emotional regulation, and long-term health trajectories [5–7]. However, the young adult developmental period is also characterized by irregular schedules, competing social and academic demands, and evolving autonomy, all of which contribute to suboptimal sleep behaviors [5,6,8].

Young adults with type 1 diabetes (T1D) may be particularly vulnerable to poor sleep health due to the complex and continuous demands of diabetes self-management, including nighttime glucose monitoring, insulin administration, and concern about nocturnal hypoglycemia [9–11]. Sleep disturbances in individuals with T1D have been associated with poorer glycemic target achievement, increased glycemic variability, impaired diabetes self-management behaviors, and greater general and diabetes-related psychological distress [12–14]. Objective and self-reported poor sleep health dimensions, including short sleep duration, low sleep efficiency, and high irregularity, are common among young adults with T1D and may exacerbate challenges in maintaining optimal glycemic targets [15–17].

Sleep is a modifiable health behavior, and improving sleep behavior often requires individuals to engage in ongoing self-regulation [18]. Self-regulation theory, grounded in Bandura’s social cognitive theory, conceptualizes behavior change as an active process involving self-monitoring, self-judgement, and self-evaluation that guides future behavior [19]. Self-regulation theory aims to explain and provide a framework for purposive decision-making by delineating the process into these three functions, which opens several different targets for behavior modification. While this framework has been widely applied to health behaviors, including substance use, physical activity, and chronic disease management [20,21], there remains a significant gap in focusing on specific conditions within defined age groups, such as young adults with T1D, who have unique social, cultural, and biological considerations.

A systematic review on the self-regulation framework of 35 chronic health interventions revealed substantial heterogeneity across different conditions, including metabolic (obese/overweight, type 2 diabetes, type 1 diabetes), cardiac (cardiac event, heart failure, coronary artery disease, cardiac rehab), arthritis, and asthma [21]. Although the three major components of self-regulation theory – self-monitoring, self-judgement, and self-evaluation—were employed across the chronic health interventions, the diversity of health behaviors (e.g., dietary changes, physical activity, medication adherence), suggests a need for a more nuanced understanding of individual differences in chronic condition management. This highlights the importance of not merely adopting a one-size-fits-all approach. Instead, healthcare systems must foster a more personalized approach accounting for the specific challenges and preferences of individuals managing chronic conditions.

However, the application of the self-regulation framework to sleep behavior—particularly in populations managing complex chronic conditions such as T1D—has not received sufficient attention. Understanding how young adults with T1D monitor, judge, and evaluate their sleep behaviors could provide critical insights into the persistent sleep difficulties they experience and inform more effective interventions. Tailoring approaches to address sleep behavior, specifically within young adults with T1D, has the potential to significantly enhance intervention planning and future efficacy.

The Sleep Behavior Self-Regulation Theory describes the set of cognitive, behavioral, and motivational processes through which individuals use to guide, adjust, and maintain their behaviors over time [22]. The Sleep Behavior Self-Regulation Theory extends general self-regulation principles to the specific context of sleep behavior and was used to guide this study [22]. Within this framework, sleep behavior self-regulation comprises three interrelated subfunctions that support effective sleep management. The first subfunction, *Sleep Behavior Self-Monitoring*, involves active observation and tracking of sleep behaviors, including bedtime routines, sleep timing, and perceived consequences such as daytime functioning or mood [22]. This active observation allows individuals to detect patterns and discrepancies between their current behavior and desired outcomes. The second subfunction, *Sleep Behavior Self-Judgement*, refers to the evaluation of one’s sleep behaviors against internal standards, societal norms, and contextual constraints, allowing individuals to determine whether their sleep practices align with personal goals or expectations [22]. The third subfunction, *Sleep Behavior Self-Evaluation*, captures the emotional and motivational responses that follow these evaluations, including feelings of satisfaction, frustration, pride, or disappointment that can either reinforce or undermine future sleep-related efforts [19,20,22]. These self-regulatory subfunctions operate dynamically and influence one another, enabling individuals to continuously adapt their sleep behaviors according to situational demands, internal cues, and environmental feedback.

These self-regulatory processes are particularly salient for young adults with T1D, whose sleep behaviors are often interconnected with diabetes-specific demands such as nighttime glucose monitoring, fear of nocturnal hypoglycemia, and technology-related alerts [16,23]. These factors may complicate bedtime routines, disrupt sleep continuity, and influence how individuals interpret and emotionally respond to their sleep outcomes. Yet little is known about how young adults with T1D make sense of these experiences or how diabetes-related considerations shape their sleep behavior self-regulation in everyday life. Qualitative descriptive methods are well-suited for capturing the lived experiences, meanings, and contextual factors that underlie sleep behavior self-regulation. Guided by Sleep Behavior Self-Regulation Theory, this qualitative descriptive study aimed to provide insights into how young adults with type 1 diabetes (T1D) monitor, judge, and evaluate their sleep behaviors through the lens of Sleep Behavior Self-Regulation Theory. The present study represents a secondary analysis of qualitative interviews originally conducted to understand barriers and facilitators of sleep among young adults with T1D (cite). This added focus allows us to address a critical gap in the literature regarding how diabetes-specific factors shape the processes underlying sleep behavior self-regulation.

## 2. Materials and Methods

### 2.1. Design

This study employed a qualitative descriptive design to capture participants’ naturalistic, plain-language accounts of their sleep behavior self-regulatory experiences. In addition to the qualitative interviews, quantitative data included clinical data collected from the chart (diagnosis, hemoglobin A1c, medications, medical history), a baseline self-report questionnaire, 7–14 days of concurrent diaries, research-grade wrist-worn actigraphy (Spectrum Plus), and continuous glucose monitoring, and a focused individual interview after the monitoring period.

Qualitative data were obtained through semi-structured focused individual phone interviews and analyzed using thematic analysis [24]. Interview duration ranged from 10 to 25 minutes depending on participant engagement and the complexity of their sleep and T1D experience. A clinician sleep report from data derived from the Spectrum Plus devices was shared with each participant and the first 5–10 minutes of the interview was spent orienting each person to their report, including a clinical impression generated by the senior author, who is a licensed clinician with specialized training in sleep and circadian research and practice (Supplemental Figure 1).

The semistructured interview guide was developed to explore young adults’ lived experiences of sleep and T1D management in the parent study. Following the clinician sleep report, the guide covered key topic domains including setting health goals based on the feedback repirt, general sleep patterns and routines, perceived barriers and facilitators, responses to sleep and diabetes disruptions.

### 2.2 Reflexivity and Bias Mitigation

All interviews and study procedures were conducted by the senior author, a licensed clinician with specialized training in sleep and circadian research and extensive prior experience conducting qualitative studies. To enhance rigor and minimize bias, the interviewer engaged in ongoing reflexive memoing throughout data collection and analysis, documenting assumptions, evolving insights, and potential analytic biases. Regular debriefing meetings with the study team were held to discuss emerging impressions, challenge interpretive assumptions, and refine interviewing techniques across participants. These safeguards provided a structured process for reflexivity and minimized the possibility that the interviewer’s clinical background or prior relationships with participants shaped data collection or interpretation.

### 2.3 Participants

Participants were recruited using a combination of MyChart push notifications, invitations from provider lists and referrals, and in diabetes speciality clinic recruitment. A detailed description of study procedures has been reported elsewhere (16,17). Briefly, young adults (18–30 years) with T1D seeking care at the Yale New Haven Health System were recruited from December 2018 to February 2020. Inclusion criteria included: having T1D diagnosis for ≥ 6 months, no diagnosis of other major chronic or psychiatric diseases, not currently enrolled in an intervention study, and being able to read or speak English language. Exclusion criteria included having previous diagnosis of obstructive sleep apnea, working night shifts, and current pregnancy. A total of 450 young adults with T1D were initially identified in the Yale New Haven Health System as potentially eligible through an EPIC electronic health system query. After screening 91 charts for eligibility, 72 were deemed eligible and invited for further screening. Of the 67 screened by phone, 46 (68%) were consented and completed the study, and 34 (74%) completed the end of study qualitative interview.

### 2.4 Procedure

The study received approval from the Yale University Institutional Review Board (2000023502). Participants were compensated $25 for completing the questionnaires, an additional $35 for returning the watch and continuous glucose monitor (CGM), and an additional $40 for participating in the exit interview. After providing their informed consent on paper, the researcher (the senior author) conducted all study procedures and interviews.

### 2.5 Analysis

We employed Braun and Clarke’s thematic analysis [24], which recognizes the researcher’s active role in identifying patterns within the data. A two-stage analytic approach was used: 1. An inductive coding approach was used to develop the preliminary codes through an independent review of transcripts and in vivo and descriptive coding to capture participants’ language and meaning without imposing a theoretical structure. Preliminary codes were compared, discussed, and refined through iterative intercoder meetings. 2. After inductive codes were established, a deductive coding approach was applied to organize the data according to the Sleep Behavior Self-Regulation Framework [22]. Inductive codes were not discarded if they fell outside the theoretical framework, but were cross-checked against all the raw data to ensure the deductive categorization did not constrain the emergence of new or divergent concepts. Thematic saturation – defined as the point at which no new codes, concepts, or meaningful content- was assessed and reached at approximately the 28^th^ interview. Interviews continued to 34 participants to ensure confirmatory depth and adequate representation across demographic subgroups. To create a more comprehensive view of the collected information, a triangulation of both quantitative and qualitative data was conducted, rather than focusing on uncovering a singular truth within the data. Coding was iterative, beginning with two coders applying inductive, *in vivo* coding to identify meaning units and refining a shared codebook through discussion. Early-stage intercoder agreement was assessed for calibration, not final reliability, and codes and themes were ultimately organized using the Sleep Behavior Self-Regulation Framework. Actiware (v. 6.0 Philips Respironics, Murrysville, Pennsylvania, United States) was used to process and analyze Spectrum Plus data collected in 30 second epochs. Glucolator 2.0 was used to process and analyze continuous glucose monitor data [25,26]. We used MAXQDA 26.0 for Mac for qualitative data organization and SPSS 31.0 for Mac for quantiative descriptive data analysis.

## 3. Results

### 3.1. Participants

Demographic and clinical descriptions of the 34 participants are presented in Table 1. Participant age ranged from 18–30 years (mean age = 22.4, SD = 3.2 years) and T1D duration ranged from 2 to 25 years (mean duration = 11.1, SD = 6.4). A majority of participants were female (n = 23, 68%), could cover their monthly expenses (97%, n = 33), and half were full-time college students (50%, n = 17). Mean hemoglobin A1C values were 7.1% (SD = 1.2%), mean CGM-derived time in range (70–180 mg/dL) was 63.3% (SD = 16.0), and mean coefficient of variation 36.5% (SD = 5.9).

Actigraphy-derived total sleep time ranged from 5 hours 24 minutes to 9 hours 25 minutes (mean duration = 7.0, SD = 1.0 hours) with 21 (62%) sleeping the recommended sleep duration (7–9 hours), 10 (29%) sleeping a shorter duration than recommended (<7 hours), and 3 (8.8%) sleeping more than recommended (>9 hours).

Sleep efficiency ranged from 73.0% to 93.0% (mean = 84.8%, SD = 4.7%), wake after sleep onset ranged from 4.2 to 72.0 minutes (mean = 37.9, SD = 17 minutes), sleep onset latency ranged from 2.9 to 59.1 minutes (mean = 19.6, SD = 13.5 minutes), and sleep variability measured by coefficient of variation of total sleep time ranged from 5.6 to 45.3% (mean = 17.8, SD = 0.09%).

Mean PROMIS sleep disturbance t-scores were 44 (SD = 8.4), mean Pittsburgh Sleep Quality Index scores were 5.6 (SD = 3.1), and mean Epworth Sleepiness Scale scores were 7.1 (SD = 3.1).

### 3.2 Overview of Themes

**Figure 1.**
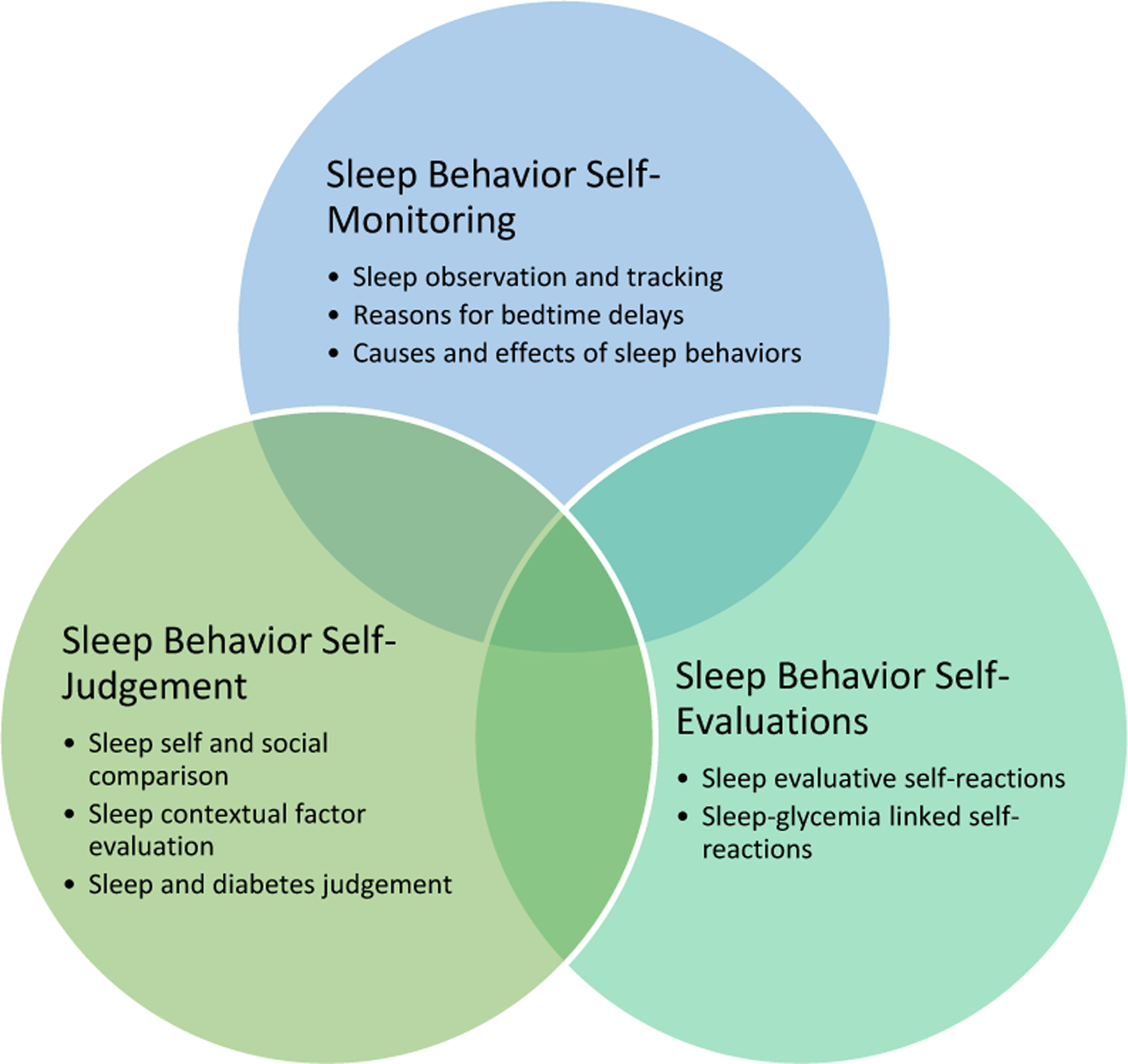
Sleep Behavior Self-Regulation Themes

Three major themes were used to organize the data: Sleep Behavior Self-Monitoring, Sleep Behavior Self-Judgement, and Sleep Behavior Self-Evaluation (Figure 1).

The *Sleep Behavior Self-Monitoring theme* included a discussion of the need to observe and track sleep habits, as well as reasons for delays in bedtime, their causes, and effects on next day function or mood. *Sleep Behavior Self-Judgement* comprised individuals’ evaluations of their sleep practices in relation to personal, societal, and contextual standards. Participants reflected on their sleep behaviors, comparing them to both self-imposed standards and societal expectations regarding healthy sleep. Various contextual factors that impacted individual sleep habits influenced this evaluation, such as work schedules, family obligations, and overall lifestyle choices. Lastly, *Sleep Behavior Self-Evaluations* were framed by participant reflections on their sleep outcomes and the emotional reactions associated with these outcomes. Participants reported experiencing both positive and negative motivations based on their sleep habits (Table 2).

### 3.3 Theme 1: Sleep Behavior Self-Monitoring

Sleep behavior self-monitoring emphasizes the importance of tracking sleep behaviors, their causes, and subsequent effects. Specifically, subthemes emerged from the focused interviews, including the need to observe and track sleep habits, reasons behind bedtime delays, causes and effects of sleep behaviors, and the distinct sleep behavior self-monitoring considerations that arise from diabetes management.

#### 3.3.1 Sleep observation and tracking

All participants interviewed were provided a sleep report with a summary (minimum, maximum, average) of their bed and waketimes, bedtime duration, sleep duration, sleep efficiency, number of awakenings, and actogram images including their rest and activity patterns and light exposure over the 7–14 days. Most participants gravitated to the images and summary statistics to reflect on their sleep patterns over the monitoring period. Many participants reported gaining knowledge from the sleep report, *“Like the blue light thing, I never really knew about that”* (P014). This was in reference to the blue light picked up by the actigraphy monitor and depicted in the report. They also described elements of the sleep environment (light, temperature, noise) and nightly routines that influenced sleep.

#### 3.3.2 Reasons for delayed bedtimes

Participants identified multiple rationales for their delayed bedtimes, including the desire to engage in stimulating activities, such as socializing, screen time, and the inevitable T1D self-management. Some explicitly identified diabetes management tasks, like blood glucose monitoring and correcting high or low blood sugars, were main contributors to their tendency to stay up later. Participants described several blood glucose instability scenarios. One participant stated, *“Good glucose control helps—highs make me restless, lows get me up”* (P019). Another participant outlined the effect glucose monitoring had on sleep quality, *“I’m watching my blood sugar that might also have an effect on [sleep quality]. So like if I’m trending high or trending low, two things that do affect me falling asleep, just sort of thinking of where I need to be*” (P008).

#### 3.3.3 Causes of sleep behaviors

The investigation into the causes of sleep behaviors identified several influential factors. Environmental conditions, such as noise and light in the sleep environment, significantly impacted participants’ ability to fall and stay asleep. Personal sleep hygiene habits, particularly increased screen time right before bed, were frequently noted as barriers to achieving restful sleep. Participants also reported experiencing general anxiety, alongside diabetes-related concerns about potential nighttime glycemic instability, which led to increased blood glucose checks, insulin administration or the need to eat, and later bedtimes.

#### 3.3.4 Effects of sleep behaviors

Participants consistently reported that the quality and quantity of their sleep directly influenced various aspects of their overall well-being, including physical health, emotional stability, and day-to-day functioning. Participants frequently commented on how having short or poor-quality sleep often made them feel more prone to getting sick, *“I think that when I don’t sleep enough, I get sick”* (P003). Furthermore, many participants attributed positive outcomes in their daytime functioning to the quality of their sleep the previous night. Those who experienced restorative sleep reported heightened levels of productivity and engagement in daily tasks. Poor sleep often resulted in fatigue and emotional strain, whereas adequate sleep supported better performance and emotional balance.

The connection between adequate sleep and next day performance and function reinforced the notion that good sleep is not just a restorative process but a critical element of functional health in managing T1D. One participant stated “*I think when my sugar is out of whack, like out of control, it really affects me because I notice that if I’m up all night with a low blood sugar or high blood sugar I’m really tired the whole next day, so that’s definitely one thing that affects my sleep*” (P010). In addition to enhanced daytime functioning, participants also linked better sleep to improved mood. Many indicated that a good night’s sleep was a precursor to feeling emotionally balanced and resilient throughout the day.

Energy levels were another significant area described as influenced by sleep behaviors. Participants noted that higher energy levels throughout the day were noticeably connected with the quality of their sleep from the night before. Good sleep provided them with the stamina needed to navigate the daily challenges of managing their diabetes, perform their tasks, and engage in social activities without feeling drained or fatigued.

### 3.4 Theme 2: Sleep Behavior Self-Judgement

The interviews demonstrated that participants evaluated their own sleep practices against personal and societal standards. This discourse introduced three critical subthemes that highlight the nuances of sleep evaluation: first, the assessment of sleep practices against personal and societal standards, which highlights the expectations and cultural norms that shaped their perceptions of healthy sleep; second, the exploration of contextual factors that influence sleep behaviors, allowing for a deeper understanding of the external circumstances that impact sleep; and third, the unique considerations for individuals with T1D, shedding light on how this specific health condition altered their sleep behavior and self-judgment.

#### 3.4.1 Evaluating sleep practices against personal or societal standards

Sleep duration was the most well-known and well-accepted standard mentioned during each of the interviews, which was consistent with the national campaign surrounding recommended sleep duration from the National Sleep Foundation [27]. The next most commonly accepted standard encompassed sleep hygiene, as participants expressed knowledge surrounding screen time and caffeine intake. Bedtime was also consistently mentioned as a consideration. Some of the lesser-known or accepted sleep practices involved stimulus control and waketime planning. For instance, several participants described spending a good deal of time spent in bed for other purposes (eating, watching TV, lounging, scrolling, etc.). Further, waketime planning (setting an alarm or making arrangements prior to the following day) was far less prevalent and seemed to be a source of anxiety brought up during several interviews.

Sleep duration was a common target for participants seeking to improve their sleep health. Most participants slept for the recommended 7–9 hours each night on average, with some sleeping too little or too much on occasion. Most deviations from the recommendation were transient rather than habitual, with one participant waking early to catch a flight on two days and another sleeping too little after staying out late. Many participants expressed goals to increase their sleep duration by going to bed earlier.

#### 3.4.2 Evaluating sleep practices through contextual factors

While personal and social expectations around sleep formed the basis of participants’ evaluations of their sleep, contextual factors played a role as well. These encompassed work schedules, environmental conditions (light, noise, temperature), and household influences. Work demands often disrupted desired sleep timing and home-life interruptions further interfered with sleep.

#### 3.4.3 Sleep and diabetes health judgement

Participants’ diabetes health emerged as a common barrier to getting good sleep. While most recognized the impact on sleep quality, one participant found more difficulty with sleep timing, *“I had like a really hard time with my blood sugar […] so I think that has definitely affected my sleep schedule”* (P030). Several participants reported waking in the night to check their blood glucose levels, *“Probably when I have to get up and check my sugars or look at my sugars. Sometimes my Dexcom is a lot of points off, like sometimes it will be 40–50 points off, so sometimes I’ll get like a little nervous, and I’ll get up to double-check”* (P032).

However, only a subset of participants offered potential solutions to diabetes-related sleep issues. One participant avoided sleeping until their blood sugar stabilized: *“I really don’t like going to bed with it either trending up or trending down, so I’ll often stay up just to make sure and manage that it’s okay”* (P006). Similarly, *“Another goal too is try to not have low blood sugar before I go to bed. Like, trying to make sure I’m stable like an hour before, so I don’t have to eat something right before I go to bed”* (P010), demonstrating awareness of the need to be more proactive about diabetes management and to set goals accordingly.

### 3.5 Theme 3: Sleep Behavior Self-Evaluations

Sleep behavior self-evaluations emerged from the participant discussions on their view of how they derive both positive motivation from personal achievements in managing their sleep and negative motivation stemming from dissatisfaction with their performance. Within this framework, two primary subthemes emerged: the reflections on sleep outcomes that evoked positive and negative motivations and the diabetes-specific affective self-reactions in response to these outcomes.

#### 3.5.1 Sleep evaluative self-reactions (positive and negative motivation)

Participants expressed surprise, concern, or motivation after reviewing their sleep data and recognizing discrepancies between perceived and actual sleep. Reviewing the sleep reports gave participants insight into their own sleep health. Occasionally, the sleep reports included positive revelations *“It wasn’t as bad as I thought it was”* (P019), though more often, the findings influenced them to modify their behavior, as one participant put it, *“Oh wow, I definitely have to make changes”* (P022).

Several participants expressed surprise with their sleep duration and number of waketimes through the night, indicating incongruence with perceived awareness of sleep duration and actual awareness of sleep duration, for example, *“But like at first, I was like there was no way I was waking up 36 times in one night. The most I remember is 3 or 4 one night when my glucose alarm went off”* (P012). Similarly, *“I’m usually a pretty sound sleeper, and I was just thinking maybe I’m not, like, as sound a sleeper as I thought”* (P009). Even though they made an effort to self-monitor their sleep, participants often overestimated their sleep duration because they conflated time in bed with total sleep time, *“I expected the total sleep time to be greater, but I guess it makes sense because the time in bed is not the same as the sleep time”* (P003).

#### 3.5.2 Sleep-glycemia linked self-reactions

Participants described how highs and lows affected sleep continuity, and how their sleep experiences influenced self-management confidence and future strategies. Participants frequently recognized the challenges and impediments to sleep quality posed by diabetes. Regarding blood glucose, one participant observed, *“When I’m high, I get more—I feel drowsy and tired, but when I’m low, I wake up—most of the time I wake up, and I can’t fall back asleep until I’m not feeling low anymore”* (P005).

### 3.6 Patterns of Convergence and Divergence in Sleep Duration and Sleep Behavior Self-Regulation

We explored patterns of convergence and divergence in sleep duration between three categories: optimal duration (7–9 hours), short duration (<7 hours), and long duration (>9 hours) across the days of monitoring. Despite differences in sleep duration, participants showed consistent engagement in core self-regulation, including tracking sleep patterns, interpreting behaviors within social and contextual factors, and linking sleep experiences to glycemic fluctuations.

Across all participants, regardless of how many hours they slept across the days of monitoring, core self-regulatory processes remained consistently present. Participants routinely tracked their sleep, interpreted their behaviors within social and contextual circumstances, and connected their nighttime experiences to fluctuations in glucose patterns, demonstrating a stable foundation of self-monitoring and diabetes-related reflection across groups.

Across groups, there was clear convergence in six subthemes spanning self-monitoring (observation/tracking; causal attributions), self-judgment (self/social comparison; contextual evaluation; sleep–diabetes judgment), and self-evaluation (glycemia-linked reactions). In contrast, three subthemes—reasons for bedtime delays, perceived effects of sleep, and general evaluative reactions—were absent among long sleepers, likely reflecting both narrative differences and the small number of participants in this group (n=3).

Notable differences across the three groups also emerged. Short and optimal sleepers discussed bedtime delays and emotional responses more frequently, offering more detailed explanations of how their sleep patterns affected daytime functioning and diabetes management. Long sleepers, by comparison, provided limited commentary on these domains, suggesting either a more generalized appraisal of sleep or reduced engagement in moment-to-moment sleep–diabetes decision-making.

When examined individually, short sleepers (mean = 6:15 h) demonstrated highly active self-monitoring (48%) and frequently described sleep delays, evaluative reflections, and diabetes-related judgment, indicating an engaged yet challenged regulatory process. Optimal sleepers (mean = 7:32 h) showed the most balanced profile, with strong self-monitoring (49%) and even distribution across judgment and evaluation (26% each). They contributed the greatest proportion of quotes reflecting detailed tracking, explanations for delays, and evaluative reactions. Long sleepers (mean = 10:04 h) emphasized self-judgment (50%) and offered few statements related to delays, consequences, or evaluations. Their limited narrative depth—combined with the small sample size—suggests cautious interpretation but indicates a regulatory style focused on broad assessments rather than detailed monitoring.

## 4. Discussion

This is the first study, to our knowledge, to investigate sleep behavior self-regulation among young adults with type 1 diabetes (T1D). We uncovered new insights into how young adults with T1D understand their sleep behaviors and the motivational processes influencing them. The findings reveal the need for a deeper exploration of sleep self-monitoring, sleep self-judgment, and sleep self-evaluation that young adults with T1D experience in relation to their sleep habits, emphasizing how their chronic condition shapes these processes. T1D presents numerous challenges surrounding sleep, and identifying areas of improvement for diabetes care and technology could vastly improve quality of life for individuals living with T1D.

Our study highlights the crucial role of active self-monitoring in managing sleep behavior, particularly among participants who identified a need to closely track their sleep patterns and their relationship to glycemic patterns. This aligns with existing literature emphasizing that self-monitoring is essential for effective chronic disease management [28]. In contrast, in a randomized crossover trial involving 32 young adults (mean age 23.8 ± 5 years) it was reported that while participants improved their nighttime sleep quality through feedback from a wearable device, their total daily sleep duration did not show significant change [29]. This raises important questions about the effectiveness of predominantly passive technology-driven interventions, which may yield mixed or null results regarding sleep health improvements [30–32]. Overall, our research underscores the necessity of incorporating active self-monitoring practices in sleep interventions to realize more significant health outcomes, particularly as reliance on passive technology may limit participants’ ability to engage meaningfully with their sleep data.

In young adults with T1D, the dynamics of sleep behavior and the self-assessment of sleep practices are critical, particularly given the considerable impact of sleep on glycemic and overall health. The recognition of societal norms around sleep, such as optimal sleep duration and hygiene, coupled with personal standards, reflects an increased awareness of the need for good sleep. However, the pressures from academic and social commitments often clashed with sleep practices of individuals in the current study, leading to feelings of frustration and guilt when they failed to meet these standards. This finding corroborates studies that have identified the influence of societal expectations on health behaviors in the general population [33,34] and in other populations with T1D [35,36]. Specifically, in a study of 619 undergraduate students followed over two years, an increased social network size was associated with lower sleep duration, and sleep levels (more or less) were influenced by their peers [37]. Increased dependence on social network sites was associated with lower perceived sleep quality and higher perceived everyday cognitive failures in a study of 340 adults (mean age = 26.11, SD = 6.54 years) [38]. In adolescents and young adults with T1D, social jet lag is associated with poorer achievement of glycemic targets [35,36]. Taken together, the expectations and individual sleep practices not only affect the well-being of young adults with T1D but also underscore the need for a deeper exploration into their emotional responses toward sleep outcomes, as these feelings significantly influence their self-regulation and health management strategies.

Sleep behavior self-evaluation revealed significant emotional responses associated with sleep outcomes, with participants describing both positive and negative motivations linked to their sleep experiences. Positive reinforcement from restful sleep was connected to feelings of accomplishment and improved emotional resilience, emphasizing the essential role of sleep in maintaining psychological well-being. In contrast, negative experiences related to inadequate sleep often led to reduced motivation and feelings of discouragement, particularly in the context of managing type 1 diabetes (T1D). These findings align with self-regulation theory, which posits that emotional reactions play a pivotal role in influencing an individual’s capacity to sustain or modify future health behavior [19,39–42]. This same mechanism has been observed across other studies focused other chronic conditions, where emotional responses to health outcomes similarly shape self-regulatory processes [39,43–46]. Indeed, studies applying self-regulation theory to adolescents and young adults managing conditions such as type 2 diabetes, cardiovascular disease, asthma, chronic pain, weight management within health behaviors, medication adherence, diet, alcohol, nicotine, and physical activity demonstrate that emotional feedback loops consistently influence health-related decision-making and behavioral follow-through. [39,43–46]. Together, this broader evidence base reinforces how the emotional reactions described by participants in the present study are not only theoretically expected but are also empirically supported across a wide range of chronic illness contexts, highlighting the central role of emotional processes in shaping sleep behavior self-regulation among young adults with T1D.

An important dimension of the sleep behavior self-regulation framework that emerged from the data is the complex relationship between diabetes management on sleep practices. Participants frequently identified the challenges posed by diabetes-related tasks that contributed to delayed sleep onset and poor sleep quality. Nighttime glucose monitoring and the fear of nocturnal hypoglycemia not only influenced sleep behavior but also became sources of anxiety that affected their ability to “switch off” at night. As such, this study underscores the need for interventions that address nighttime glucose stability. Other studies reiterate these findings about patient experiences with sleep and T1D. Prior research has established the interdependent, cyclical relationship of sleep and diabetes, wherein the worsening of one can worsen the other. Emotional distress regarding blood glucose management at night and nighttime hypo- or hyperglycemia emerged as the most disruptive element of T1D management in participants across studies [47–50]. Several other factors pertaining to both sleep and diabetes also appear in the literature. The mixed role of technology in sleep has been reported in other studies, both as a comfort in glucose monitoring and as a disruptor in many of the same ways described by participants in this study [47–51]. The literature also reinforces the benefits of exercise for sleep, particularly within the context of T1D, yet individuals in other studies were often reported not to meet the recommended levels of physical activity [48].

Self-regulation, defined by a set of cognitive, behavioral, and emotional strategies, positively correlates with glycemic outcomes and T1D management, particularly in youth and young adult populations [41,42,52]. These strategies allow individuals to modulate their responses to stressors and increase self-efficacy [39]. Self-regulation has a protective effect in the presence of major life challenges, such as a global pandemic, specifically in populations with T1D [52]. Based on the findings from this study, individuals with high levels of self-regulation manage their sleep and diabetes health more effectively by enforcing good sleep hygiene, committing to regular exercise, managing stress, and proactively addressing diabetes-related issues.

There are several limitations to consider in the context of the current study. First, this is a secondary analysis of existing data over a 2-week monitoring period, and the primary purpose of the study was not to explore sleep behavior self-regulation. This could be strengthened in future studies by designing a more comprehensive interview guide, ideally situated within a longitudinal non-experimental or experimental design to explore the nuances of sleep behavior self-regulation and how this phenomenon changes over time. Second, data were collected from a single health system in the Northeast, a majority of the sample were women (70.6% vs. 50.9% distribution in the US [53]) who could cover their monthly expenses, without other major chronic co-morbid medical or major psychiatric conditions, thus limiting generalizability of the findings. Due to the fact that participants were recruited through outpatient diabetes clinics and patient portals, the sample may disproportionately reflect young adults who are more engaged in routine diabetes care, have more stable living circumstances, and may have greater access to diabetes technologies such as CGM. These contextual factors likely shape both their sleep behaviors and their broader self-regulatory processes. Strengths of the study include the rich, in-depth data derived from the individual focused interviews alongside objective sleep behavior data and strict inclusion criteria. It should be noted that the intention of qualitative inquiry is not generalizability and the control of covariates and confounders inherent in the parent study design reduced selection bias and improved internal consistency.

From a clinical perspective, the insights from this qualitative inquiry can inform the development of tailored interventions to promote healthy sleep opportunities for young adults with T1D. The integration of sleep behavior self-regulatory strategies within diabetes care could be the way to assess multimodal sleep behaviors. Future research should explore the effectiveness of such integrated approaches with digital health-supported multimodal sleep behavior interventions to enhance both sleep regulation and diabetes management. For instance, digital health technologies, as a format for behavioral interventions, have been effective in many areas in improving target outcomes, such as adherence to healthy behaviors [54–61]. CGM use is also expanding beyond outpatient care, with growing evidence supporting its utility in inpatient and other specialized settings to improve detection of glycemic variability and guide timely clinical decisions. Incorporating sleep-relevant metrics into CGM-supported workflows may help clarify how disrupted sleep interacts with glycemic patterns during hospitalization or acute care episodes and inform more holistic, context-specific intervention strategies.

This study highlights the complex relationship between sleep behavior self-regulation and the management of type 1 diabetes among young adults. Participants indicated that their monitoring and evaluation of sleep directly influence their diabetes management and overall health. Healthcare providers should therefore implement targeted digital health interventions that combine sleep education with diabetes care, such as mobile apps that simultaneously track sleep hygiene and blood sugar levels. This approach could empower young adults to optimize their sleep routines. Additionally, addressing emotional responses to sleep outcomes could enhance motivation and resilience. Future research should focus on evaluating the effectiveness of these integrated interventions and leveraging digital technology to support self-regulation in both sleep and diabetes management.

## Supplementary Material

Supplemental Figure 1. Sample Clinician Sleep Report

**Supplementary Materials:** Supplemental Figure 1. Sample Clinician Sleep Report.

## Figures and Tables

**Table 1.** Individual participant demographics and clinical characteristics

ID	Sex	Age	Sleep Duration (hours)^a^	A1C^b^	Time in Range %^c^	ID	Sex	Age	Sleep Duration (hours)^a^	A1C^b^	Time in Range %^c^
**1**	F	28	8:30	5.80	73.20	**18**	F	26	6:10	6.20	86.03
**2**	M	21	7:56	6.30	72.99	**19**	F	27	7:17	6.10	86.66
**3**	F	22	7:20	6.70	59.66	**20**	F	25	6:31	7.70	77.53
**4**	M	23	6:15	8.10	56.72	**21**	F	19	8:47	7.40	54.52
**5**	F	21	8:22	6.80	40.80	**22**	M	29	6:21	5.60	94.90
**6**	F	29	8:59	7.50	51.19	**23**	M	19	7:30	6.80	57.05
**7**	M	22	9:57	9.40	30.55	**24**	F	22	8:45	8.20	37.52
**8**	F	24	7:25	6.80	70.48	**25**	M	21	6:59	7.20	77.06
**9**	F	25	7:59	6.80	55.97	**26**	M	18	6:22	5.50	75.49
**10**	F	24	6:31	6.50	81.75	**27**	M	18	6:41	7.60	57.56
**11**	F	19	5:57	8.10	48.67	**28**	F	23	7:33	6.30	48.11
**12**	F	25	7:52	7.10	70.16	**29**	F	18	8:55	8.30	49.74
**13**	F	22	7:00	4.70	52.78	**30**	F	19	7:28	9.00	61.22
**14**	F	19	10:12	6.50	64.84	**31**	F	23	8:47	8.00	45.69
**15**	M	20	7:54	6.40	79.33	**32**	F	25	6:37	9.10	52.75
**16**	F	22	10:06	9.50	*	**33**	M	21	7:26	7.20	57.53
**17**	M	19	7:06	5.80	91.07	**34**	F	25	7:04	6.90	69.31

* Missing data.

a Actigraphy-derived sleep duration in hours across monitoring days.

b Hemoglobin A1C from chart extraction. Continuous glucose monitor-derived percentage of time in range, 70–180 mg/dL across monitoring days.

**Table 2.** Themes, Subthemes, and Illustrative Quotes

Theme	Subtheme	Illustrative Quotes
**Theme 1: Sleep Behavior Self-Monitoring**	**Sleep observation & tracking**	“I try to keep…track of my sleep…and how many hours I’m usually getting.” (P004) “I never really knew about [the blue light] until I saw it in the report.” (P014)
	**Reasons for delayed bedtimes**	“I’m bad about watching TV or playing on my phone and usually fall asleep doing one of those.” (P028) “Had an 8 am class, went to bed at 12:15 a.m.” (P002)
	**Causes of sleep behaviors**	“I go to the bathroom brush my teeth go to the bathroom, and then go to my room and then I just turn off the lights in my room it’s really dark in my room and then go to sleep and normally like 95% of the time I will fall asleep within like 5 minutes” (P026). “If I have any caffeine past 3 o’clock, I have difficulty sleeping.” (P009) “If I nap during the day, I wake up at 3 a.m. fully energized.” (P029)
	**Effects of sleep behaviors**	“If I’m in bed before 1 a.m., the next day is set.” (P017) “If I’m up all night with low or high blood sugar, I’m really tired the whole next day.” (P010)
**Theme 2: Sleep Behavior Self-Judgment**	**Personal/social standards**	“I need to go to sleep earlier and not be on my phone before bed.” (P017) “Checking my phone at night probably isn’t the best.” (P016)
	**Contextual factors**	“I’m trying to change my shift to have a more consistent bedtime.” (P019) “Light wakes me up…we have blackout blinds now.” (P019) “I need dark and quiet and, you know, a good temperature” (P005)
	**Sleep–diabetes judgment**	“High or low blood sugar wakes me up at night.” (P016) “I don’t like going to bed when it’s trending up or down—I stay up to make sure it’s okay.” (P006)
**Theme 3: Sleep Behavior Self-Evaluation**	**Evaluative self-reactions**	“It wasn’t as bad as I thought it was.” (P019) “Oh wow, I definitely have to make changes.” (P022)
	**Sleep-glycemia linked emotional reactions**	“When I’m high, I feel drowsy; when I’m low, I wake up and can’t fall back asleep.” (P005) “I forget to check my blood sugar sometimes…I should be better about it.” (P030)

**Table 3.** Group Level Joint Display of Sleep Duration and Qualitative Evidence

Sleep Duration Group^a^	Short Duration	Optimal Duration	Long Duration
**Key Characteristics**	Highly active, challenged self-regulation	Most balanced self-regulation profile	Generalized, less elaborated self-regulation
**Convergence**	Short sleepers discussed more bedtime delays, emotional responses, and diabetes-driven disruptions—patterns that align with what one would expect in individuals obtaining insufficient sleep	Optimal sleepers displayed the highest volume of regulatory behaviors across monitoring, judgment, and evaluation:	Long sleepers… provided fewer statements… absence of discussion of bedtime delays… minimal emotional evaluations… sparse detail across subthemes
**Divergence**	Short sleepers… demonstrated notably active Self-Monitoring… clustered around observation, tracking, and self/social comparison	Optimal sleepers contributed the largest share of quotes related to tracking, reasons for delays, and evaluative self-reactions	Long sleepers gave almost no commentary about how sleep affected next-day functioning

Note: Optimal 7–9 hours; Short <7 hours; Long >9 hours. ^a^

## Data Availability

The data used for this study is available on reasonable request to Dr. Stephanie Griggs at stephanie.griggs2@emory.edu

## Abbreviations

T1D: Type 1 Diabetes
A1C: Glycated hemoglobin
