# Supplementary material for: A Thematic Analysis of Sleep Behavior Self-Regulation in Young Adults with Type 1 Diabetes
Source: Diabetology (Basel). Author manuscript; Available in PMC 2026 May 9. (PMC13155417; doi:10.3390/diabetology7040080)
Supplement: Supplemental Figure 1. Sample Clinician Sleep Report [file NIHMS2167095-supplement-Supplemental_Figure_1__Sample_Clinician_Sleep_Report.docx]

**Supplemental Figure 1.** Sample Clinician Sleep Report


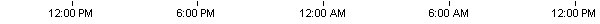

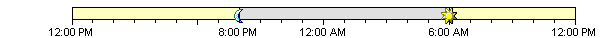

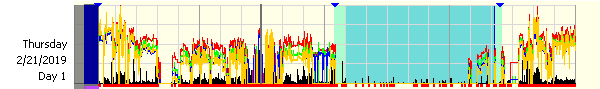

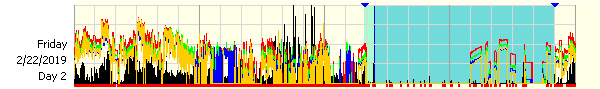

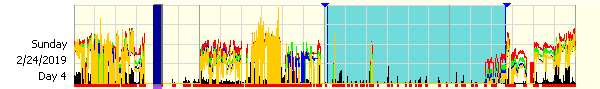

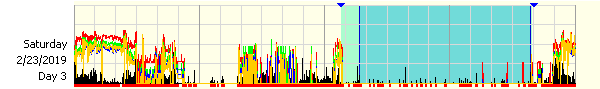

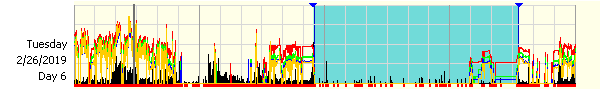

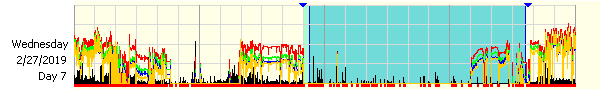

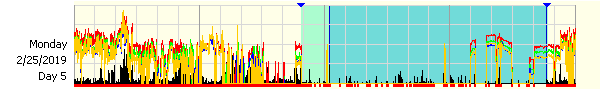

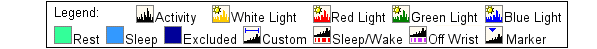


**Actogram:**

**Activity Scale:**

ID: 009

Summary Statistics:

|  | Bed Time | Get Up Time | Time in Bed (hours) | Total Sleep Time (hours) | Onset Latency (minutes) | Sleep Efficiency (percent) | WASO (minutes) | #Awak. |
| --- | --- | --- | --- | --- | --- | --- | --- | --- |
| Min | 10:53:00 PM | 7:05:30 AM | 7:12:00 | 6:14:30 | 2.50 | 74.54 | 32.50 | 30 |
| Max | 1:55:00 AM | 11:02:30 AM | 11:47:00 | 8:54:00 | 82.00 | 90.66 | 97.50 | 66 |
| Avg | 12:03:11 AM | 9:21:11 AM | 9:18:00 | 7:47:26 | 29.44 | 84.11 | 56.75 | 47.12 |
